# Adding Extracorporeal Membrane Oxygenation to Cardiopulmonary Resuscitation in Out-of-Hospital Cardiac Arrest Due to Pulmonary Embolism: A Case Report

**DOI:** 10.7759/cureus.52443

**Published:** 2024-01-17

**Authors:** Rui Caetano Garcês, Raquel Avelãs Cavaco, Philip Fortuna, Luís Bento

**Affiliations:** 1 Unidade de Urgência Médica, Centro Hospitalar Universitário de Lisboa Central, Lisbon, PRT

**Keywords:** out-of-hospital cardiac arrest, complications of anticoagulation, systemic thrombolysis, venoarterial extracorporeal membrane oxygenation, massive pulmonary embolism

## Abstract

We present a challenging cardiopulmonary resuscitation scenario of an out-of-hospital cardiac arrest (OHCA) in a 21-year-old healthy woman recovering from a lower limb fracture who collapsed during a rehabilitation session at a community center. The combination of witnessed arrest, administration of immediate cardiopulmonary resuscitation, and effective communication to emergency services enabled a timely cannulation of extracorporeal membrane oxygenation in a cardiopulmonary resuscitation reference center. The cause of the cardiac arrest was pulmonary embolism, and the intensive care unit team opted for thrombolysis when she arrived after 40 minutes of cardiopulmonary resuscitation. The circulatory support given by venoarterial extracorporeal membrane oxygenation enabled adequate perfusion until the restoration of cardiac blood flow at 75 minutes after cardiac arrest. Despite the initial success, several life-threatening complications occurred. Anticoagulation is of paramount importance during extracorporeal support, as is thrombolysis in massive pulmonary embolism with cardiac arrest. However, this led to several complications. We highlight the importance of liaising with a wider team in such cases, including hepatobiliary surgery, vascular surgery, and interventional radiology, as doing so saved this patient’s life without deficits.

## Introduction

Out-of-hospital cardiac arrest (OHCA) presents a formidable challenge in emergency medicine. Not only is it commonly refractory to conventional cardiopulmonary resuscitation (CPR) with a disheartening survival rate of 8% but it is also associated with adverse neurological outcomes in survivors [1]. In this complex landscape, the integration of extracorporeal membrane oxygenation (ECMO) into CPR, known as eCPR, has emerged as a promising intervention to improve these outcomes [2]. Studies have begun demonstrating the potential of eCPR to offer superior survival and neurological outcomes compared to conventional CPR alone in patients with initial presentation with ventricular fibrillation and ventricular tachycardia rhythm [3-7]. However, specific data on the effectiveness of eCPR in cases of OHCA because of massive pulmonary embolism (PE) remain limited [8,9], highlighting a critical gap in current medical knowledge and practice. Our case report aims to contribute to this emerging field, drawing upon advanced strategies as discussed by Yannopoulos et al. [10] and the cost-effectiveness analysis by Dennis et al. [11]to provide a comprehensive view of the challenges and opportunities in the application of eCPR for OHCA because of PE.

## Case presentation

We present the case of a 21-year-old woman with an unremarkable medical history who, three weeks before this case report, had a right tibial fracture that was treated with intramedullary nailing surgery. She completed two weeks of deep vein thrombosis prophylaxis with apixaban and was being physically rehabilitated at a community center that happened to have an anesthetist on its staff. During a regular session of physiotherapy, she sustained a witnessed cardiorespiratory arrest with an immediate start of CPR from a differentiated healthcare professional (anesthetist).

The prehospital emergency services were activated, and, upon its arrival, an initial rhythm of pulseless electrical activity (PEA) was documented. Advanced cardiovascular life support was initiated, which included the Lund University Cardiac Assist System or LUCAS® 3 (Stryker Corporation, MI), and the patient was handed over by the doctor from the community center.

Considering the presentation and potential for reversibility, an intensive care unit referral center for eCPR was contacted.

This patient met all the criteria of eligibility for eCPR at this Center with an age inferior to 60 years old, time without CPR inferior to five minutes, and entrance in ECMO achievable in less than 60 minutes in an otherwise previously healthy patient. Therefore, she was accepted and transported to the hospital.

Upon her arrival, 40 minutes postarrest, with persistent PEA and high clinical suspicion of PE, alteplase administration was initiated first with a 50 mg bolus, followed by a 50 mg infusion. Simultaneously, venoarterial (VA)-ECMO cannulation was performed with the introduction of a 17 Fr femoral arterial cannula and a 25 Fr femoral venous cannula. Extracorporeal circulation was established 57 minutes postarrest, and the initial ECMO settings were a blood flow of 3.3 L/min from 2600 rotations per minute (rpm) and a sweep gas flow of 2 L/min. The positioning of the cannulas was confirmed with ultrasound, and the venous cannula was positioned 2 cm from the suprahepatic veins. The center anticoagulation protocol states a bolus of unfractionated heparin in the entrance of extracorporeal circulation that was not given because of concurrent fibrinolysis treatment, afterwards, an infusion of heparin was initiated at a rate of 40 units/kg/h that was monitored with point-of-care partial thromboplastin time (aPTT).

**Figure 1:**
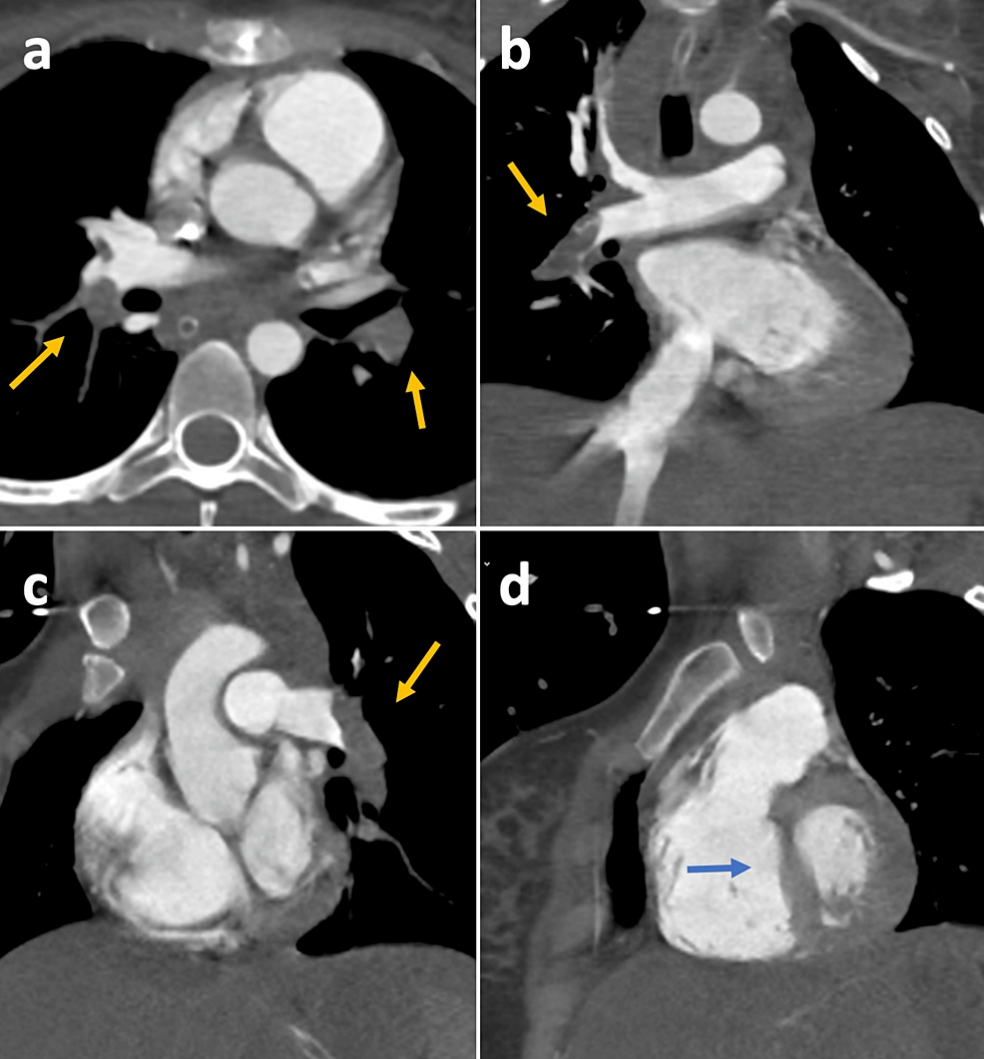
CT pulmonary angiogram with signs of acute PE Axial (a) and coronal oblique images (b,c) depicting multiple obstructive filling defects (yellow arrows); coronal oblique image (d) showing indirect signs of pulmonary hypertension, namely, right ventricular dilatation and interventricular septum flattening (blue arrow).

At 75 minutes from the initial arrest, cardiac blood flow was recovered. Even with ECMO support and thrombolytic therapy, echocardiography displayed continued right ventricular dilation indicative of severe strain, and CTPA confirmed a substantial central PE, with additional identification of hepatic and splanchnic hematomas, which are likely attributable to the resuscitative efforts. The presence of femoral deep vein thrombosis was affirmed by Doppler ultrasound. Neurologically, she exhibited responsive pupils and withdrawal reflexes to noxious stimuli while sedated. CTPA images at the time of her admission are shown in Figure 1.

At six hours post-admission and fibrinolysis, the patient subsequently developed an acute abdomen with voluminous hemoperitoneum and a hepatic subcapsular hematoma of segments II and III, progressing rapidly into severe hemorrhagic shock refractory to volume and massive transfusion protocol with 10 units of blood. During the period of hemorrhagic shock, anticoagulation and fibrinolysis were reverted, as guided by rotational thromboelastometry ROTEM® (TEM International, Munich, Germany), with five pools of fresh frozen plasma, one pool of platelets, 2 g of tranexamic acid, and 5 g of fibrinogen.

**Figure 2:**
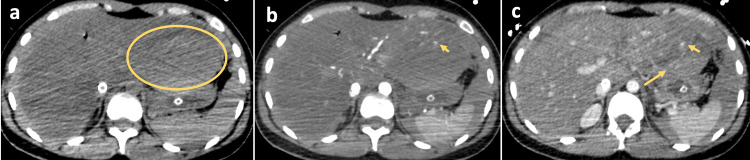
Abdominal computed tomography scan before and after the administration of iodine intravenous contrast medium (a) Non-enhanced axial image showing a spontaneously dense hematoma between the left lobe of the liver and the stomach; enhanced axial images in arterial (b) and portal (c) phases showing active bleeding (arrows) and progressive increase of hematoma density.

An abdominal computed tomography (CT) scan with iodine intravenous contrast medium was repeated, which revealed a significant increase in blood collection located between the left lobe of the liver and the stomach (97x62 mm), suggestive of subcapsular hematoma, with a focus of active hemorrhage inside, apparently originating from the left hepatic artery, and doubtful foci coming from the short gastric (Figure 2). The voluminous hemoperitoneum was dispersed perisplenic, perihepatic, along the paracolic gutters, and in the pelvic cavity.

Considering the refractory shock with suspicion of arterial abdominal hemorrhage and PE with right ventricle failure, the patient was submitted to thrombectomy and hepatic artery embolization executed by interventional radiology 12 hours post-admission. After these interventions, ECMO support was successfully weaned, and, on the second day post-admission, the patient was decannulated. This procedure was complicated with local hemorrhage requiring a vascular surgery review. As an attempt to close the superficial femoral artery with suture-mediated closure using the Perclose ProGlide® System (Abbott Vascular Inc., Santa Clara, CA) was unsuccessful, it was placed in a Gore® DrySeal Flex Introducer Sheath (W. L. Gore & Associates, Newark, DE) and direct arteriography of the superficial femoral artery, and thrombectomy was done with a Fogarty occlusion catheter (Edwards Lifesciences, Irvine, CA), plus direct venography of the common femoral vein. She also received an inferior vena cava filter because of persistent lower limb venous clots, on the same day.

After clinical stabilization, anticoagulation was reinstated on the sixth day of hospitalization, beginning with unfractionated heparin infusion with an aPTT ratio goal of 1,5. She remained uneventful, and, on the tenth day, she was switched to enoxaparin 40 mg twice daily with tight anti-Xa activity control. Despite an initially good response, on the 16th day of hospitalization, she developed a sustained drop in hemoglobin and worsening of the hepatic hematoma documented by CT scan. For that, the hepatobiliary surgery department decided that the best course of action would be a left hepatic lobectomy via median laparotomy that was performed later that day.

After requiring five days of mechanical ventilation and 14 days of renal replacement therapy for cortical necrosis, multiple complications of her cardiopulmonary arrest, and ensuing treatment challenges, she was able to achieve full neurological recovery. Subsequently, she progressed to the high-dependency unit for continued recovery.

On admission to the HDU, the patient developed a rapidly progressive oral intolerance and anemia with Hb=5.3 g/dL. The abdominal CT showed, at the lobectomy site, a hematic collection measuring 131x69x38 mm (198 mL) with a larger cephalocaudal axis. In posterior continuity with the stomach and laterally beside it, a semiologically similar collection with 142x103x60 mm (456 mL) was seen. Lateral to the latter, a third collection was found measuring 103x84x47 mm (217 mL). She had previously restarted 1 mg/kg of enoxaparin with tight anti-factor Xa activity control that had always been within the therapeutic range. Treatment with enoxaparin was halted, and the patient was managed conservatively with cessation of anticoagulation and supportive care including blood products. Repeated CT scans showed improvement, and anticoagulation was restarted on the 32nd day of hospitalization, with unfractionated heparin later converted to enoxaparin. In total, the patient received 31 units of packed red cells from admission to discharge.

The inferior vena cava filter was removed with no complications on the 43rd day. Repeat imaging showed a residual mural clot on the popliteal vein, and a persistent right thigh hematoma measuring 63x18 mm was managed conservatively.

After a complex initial presentation, the patient recovered rapidly, demonstrating improved exertional tolerance and no longer requiring supplemental oxygen. Follow-up echocardiography showed resolution of right-sided heart dilation, with a tricuspid annular plane systolic excursion (TAPSE) of 26 mm and an estimated pulmonary artery systolic pressure (PASP) of 25 mmHg.

## Discussion

This case illustrates a successful intervention for an OHCA resistant to conventional CPR, navigating through a spectrum of complications to overturn a generally poor prognosis.

This was enabled by a succession of factors. First, both the duration of time without CPR (no-flow) and the duration of basic life support and advanced cardiovascular life support (low-flow CPR) were minimized. Second, it was a witnessed collapse with timely CPR intervention executed by a differentiated healthcare professional. The handover to the pre-hospital medical team was also critical, as it was not only quick but enabled the presumption of the likely cause of cardiac arrest, its reversibility, and an early activation of the eCPR team.

This combination of factors led to a timely ECMO cannulation and entrance to extracorporeal life support (high-flow CPR) at 57 min from the cardiac arrest. Despite the initial success in regaining flow, tissue perfusion, and, afterward the return of spontaneous circulation, several life-threatening complications ensued.

Systemic anticoagulation and thrombolysis were required for the massive PE and maintenance of VA-ECMO. Although fundamental, those therapeutical interventions led to several hemorrhagic complications that required liaising with a wider team to save this patient’s life.

The hospital's tertiary care infrastructure enabled a multidisciplinary approach, fostering collaboration between surgical specialties such as hepatobiliary and vascular surgery, along with interventional radiology, which was vital for the patient's survival.

OHCA affects 275,000 individuals in Europe annually [12], and it is associated with high mortality and morbidity. Approximately 90% of these patients do not survive to hospital discharge. Even with high-quality advanced life support, after 35 minutes of resuscitation, only 1% survive without neurological impairment [13].

Implantation of VA-ECMO provides respiratory and circulatory support and has been used in both in-hospital cardiac arrest (IHCA) and OHCA. Evidence has mounted showing improvement in survival with favorable neurological outcomes in adults with refractory OHCA, first in observational studies and recently in high-quality randomized clinical trials. The use of eCPR for refractory OHCA has been associated with survival rates of 6.9%-55.0% depending on the selection criteria of different studies [3,6,7].

One of the common concerns regarding eCPR is its cost and cost-effectiveness. However, a Markov model developed using the data from two ECMO centers in Sydney, Australia, to evaluate the cost-effectiveness of eCPR for IHCA and OHCA, found it to be cost-effective in terms of cost per quality-adjusted life year for Australia, Europe, and the United States [11].

Although most data for eCPR come from cohorts (i.e., cohort studies), the ARREST trial - a phase-2, single-center, open-label, randomized, controlled study of ECMO-facilitated resuscitation in patients presenting witnessed OHCA with an initial rhythm of ventricular fibrillation or pulseless ventricular tachycardia - was reported in *The Lancet *during 2020 [10]. This RCT concluded that early ECMO-facilitated resuscitation in patients with refractory shockable OHCA significantly improved survival to hospital discharge when compared to standard ACLS treatment (43% vs 7%).

While ECMO is established in the management of cardiogenic shock because of massive PE, its specific role in eCPR for OHCA secondary to PE is not well-documented, presenting an area for further research. On the one hand, with the growing availability of ECMO for OHCA, it is expected that PE would be considered for inclusion. On the other hand, there are many unknowns, including whether ECMO improves the survival rate of these patients at all. Additionally, it is also unknown whether thrombolysis or embolectomy should additionally be used with ECMO in patients with PE [8].

A recent retrospective study of a 13-year German database found that from the 1,172,354 patients hospitalized with PE; 2,197 (0.2%) were treated with ECMO support. Cardiac arrest requiring CPR was present in 77,196 (6.5%) patients. Although more than one-fourth of those patients were treated with systemic thrombolysis alone (n = 20,839 patients; 27.0%), a minority of patients received thrombolysis and VA-ECMO (n = 165; 0.2%), embolectomy and VA-ECMO (n = 385; 0.5%), or VA-ECMO alone (n = 588; 0.8%). A multivariable logistic regression analysis indicated the lowest risk for in-hospital death in patients who received embolectomy in combination with VA-ECMO (OR = 0.50; 95% CI = 0.41-0.61; p < 0.001), thrombolysis and VA-ECMO (OR = 0.60; 95% CI = 0.43-0.85; p = 0.003), or VA-ECMO alone (OR = 0.68; 95% CI = 0.57-0.82, p < 0.001) compared to thrombolysis alone (OR = 1.04; 95% CI = 0.99-1.01; p = 0.116). Hence, these results suggest that the use of VA-ECMO alone, or as part of a multipronged reperfusion strategy including embolectomy or thrombolysis, might offer survival benefits compared to thrombolysis alone in patients with PE deteriorating to cardiac arrest [9].

These findings resonate with our case, where the patient, suffering from OHCA due to PE, also benefited from the rapid administration of thrombolytics followed by ECMO support. Despite the differences in setting (IHCA versus OHCA), the positive outcomes in the subgroup provide a compelling argument for the application of this strategy in OHCA cases with similar clinical presentation.

## Conclusions

Further research is needed in this subgroup of patients with OHCA secondary to PE to establish best care practices regarding both VA-ECMO but also reperfusion strategy (anticoagulation, thrombolysis, and/or thrombectomy). In this patient’s case, the initial approach was successful, but a series of complications arose that required expert care from both vascular surgery and interventional radiology.

eCPR has the potential to enhance patient outcomes significantly. However, after implementation, it should be continued within referral centers equipped to address the potential complications and provide comprehensive extracorporeal organ support. The efficacy of critical care interventions, such as eCPR, hinges on the availability of dedicated multidisciplinary teams that can collaborate seamlessly across various specialties. The case presented demonstrates a favorable outcome for a young patient, largely owing to the comprehensive care provided by the medical team, and her access to specialized resources. This outcome serves to highlight the crucial role of resource availability and multidisciplinary care in managing complex critical conditions effectively.
